# Reduction in cortical gamma synchrony during depolarized state of slow wave activity in mice

**DOI:** 10.3389/fnsys.2013.00107

**Published:** 2013-12-16

**Authors:** Eunjin Hwang, James M. McNally, Jee Hyun Choi

**Affiliations:** ^1^Center for Neuroscience, Korea Institute of Science and TechnologySeoul, South Korea; ^2^VA Boston Healthcare System, Department of Psychiatry, Harvard Medical SchoolBrockton, MA, USA; ^3^Department of Neuroscience, University of Science and TechnologyDaejon, South Korea

**Keywords:** gamma oscillations, slow wave activity, electroencephalography (EEG), local field potential (LFP), phase synchronization, ketamine anaesthesia

## Abstract

EEG gamma band oscillations have been proposed to account for the neural synchronization crucial for perceptual integration. While increased gamma power and synchronization is generally observed during cognitive tasks performed during wake, several studies have additionally reported increased gamma power during sleep or anesthesia, raising questions about the characteristics of gamma oscillation during impaired consciousness and its role in conscious processing. Phase-amplitude modulation has been observed between slow wave activity (SWA, 0.5–4 Hz) and gamma oscillations during ketamine/xylazine anesthesia or sleep, showing increased gamma activity corresponding to the depolarized (ON) state of SWA. Here we divided gamma activity into its ON and OFF (hyperpolarized) state components based on the phase of SWA induced by ketamine/xylazine anesthesia and compared their power and synchrony with wake state levels in mice. We further investigated the state-dependent changes in both gamma power and synchrony across primary motor and primary somatosensory cortical regions and their interconnected thalamic regions throughout anesthesia and recovery. As observed previously, gamma power was as high as during wake specifically during the ON state of SWA. However, the synchrony of this gamma activity between somatosensory-motor cortical regions was significantly reduced compared to the baseline wake state. In addition, the somatosensory-motor cortical synchrony of gamma oscillations was reduced and restored in an anesthetic state-dependent manner, reflecting the changing depth of anesthesia. Our results provide evidence that during anesthesia changes in long-range information integration between cortical regions might be more critical for changes in consciousness than changes in local gamma oscillatory power.

## Introduction

Defining the mechanisms which underlie consciousness represents a vital unresolved question in neuroscience (Brown et al., 2010). Changes in conscious state involve dramatic shifts in neural activity, including changes in the level of oscillatory rhythms. Such oscillations, particularly within the gamma frequency range (30–100 Hz), have been suggested to represent an essential mechanism to provide temporal coordination of neuronal activity across local and distributed neural regions. Thus, gamma oscillations are believed to play a central role in feature binding, allowing coherent integration of multisensory input at a millisecond scale (Gray and Singer, 1989; Tallon-Baudry and Bertrand, 1999; Engel and Singer, 2001), and are required for a number of cognitive processes including selective attention and both long and short term memory formation (Lisman, 1999; Buzsaki and Draguhn, 2004). Given that such processes perhaps reflect the functional basis of consciousness, gamma activity has been hypothesized by many to represent a direct correlate of consciousness (Massimini et al., 2012).

Contrasting this idea, several recent studies have noted gamma activity can also be observed during both slow-wave sleep and anesthesia (Steriade, 2006; Chauvette et al., 2011; Valderrama et al., 2012), when consciousness is believed to be reduced. Interestingly, this gamma activity has been observed to be nested in the depolarizing (ON) phase of slow wave activity (SWA). SWA is believed to originate from neocortical neuronal networks (Steriade et al., 1993; Timofeev and Steriade, 1996; Steriade and Pare, 2007). However, thalamocortical relay neurons may also participate in their generation, by triggering the cortical depolarization resulting in onset of the ON state (Destexhe et al., 2007; Crunelli and Hughes, 2010). The ON state of SWA has been found to share a number of similarities with wake state activity, including electrophysiological features such as behavior of neuronal membrane potential and firing rate (Destexhe et al., 2007; Constantinople and Bruno, 2011). In this context, the observation of nested gamma activity during this phase of SWA raises the question of whether this gamma activity has similar properties to that observed during wake.

Here we directly compare the properties of gamma oscillations observed during the ON state of SWA to those observed during wake. Ketamine/xylazine anesthesia was used as a model for the SWA since it leads to EEG pattern with alternation of depolarized state and hyperpolarized state which have similar properties to those occurring during natural sleep (Steriade et al., 1993; Contreras and Steriade, 1995). In this work, electroencephalogram (EEG) and local field potential (LFP) activity was recorded from several thalamocortical regions including motor-related and somatosensory-related cortical regions, located in frontal and parietal cortex, respectively, in addition to the thalamic nuclei which have anatomical connections to the corresponding cortical regions. Gamma oscillatory activity observed during ketamine/xylazine anesthesia was divided into ON state gamma and OFF (hyperpolarizing) state gamma, and then compared to the baseline levels during wake. The region-specific changes of gamma power and synchrony were calculated throughout anesthesia and consecutive recovery, and the values were compared across the states with different depth of anesthesia. The ON state gamma power remained at the same level compared to wake, however, the synchrony between somatosensory and motor cortex decreased significantly.

## Materials and methods

### Ethics statement

All the mice used in this research were treated according to the Act 1992 of the Korea Lab Animal Care Regulations and Associated Guidelines. All the surgical and experimental procedures for electrophysiological recording and anesthesia were approved by the Institutional Animal Care and Use Committee in Korea Institute of Science and Technology.

### Animal preparation and surgery

**Figure 1:**
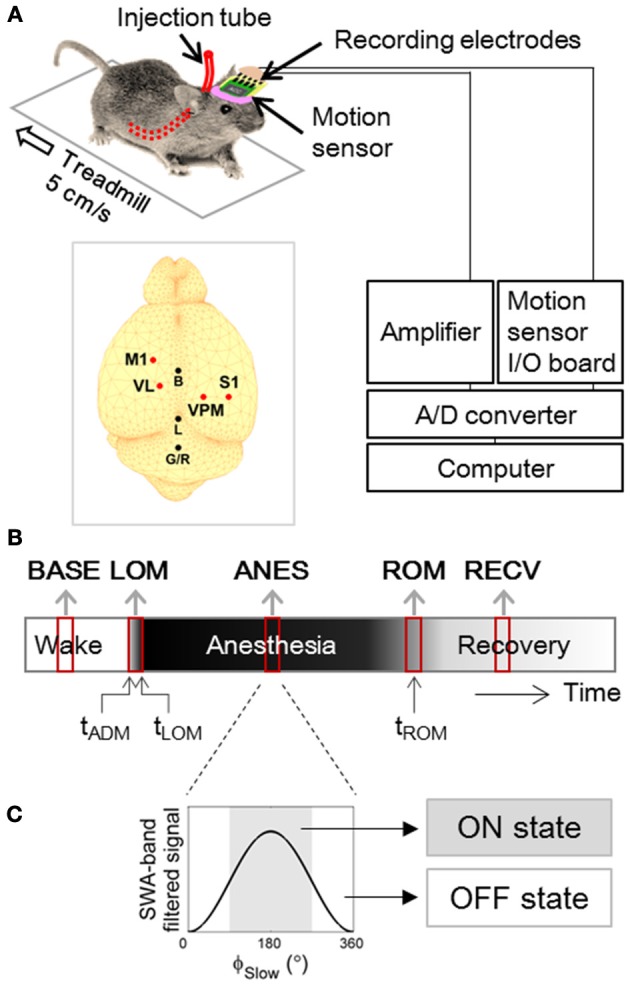
**Experimental Design. (A)** Experimental setup for forced walking test (Hwang et al., 2010). Inset figure shows locations of screw and wire electrodes for EEG/LFP recordings. M1, primary motor cortex; S1, primary somatosensory cortex; VL, ventral lateral thalamic nucleus; VPM, ventral posteromedial thalamic nucleus; B, bregma; L, lambda; G/R, ground and reference. **(B)** Timeline of EEG and LFP recording. Two to three min of artifact-free steady periods were sampled from baseline (BASE), anesthesia (ANES), and motion-recovered (RECV) state, and transitional periods were sampled around the moment of loss of motion (LOM) and the moment of recovery of motion (ROM). **(C)** Anesthetic period was sub-classified based on the phase of slow wave (ϕ_Slow_) dominant during anesthesia, into ON state where 90° ≤ ϕ_Slow_ <270° and OFF state where ϕ_Slow_ <90° or ϕ_Slow_ ≥270°. ϕ_Slow_ was calculated by Hilbert transformation of EEG/LFP signal filtered between 0.5–4 Hz (400th order zero-phase FIR band-pass filter).

Male C57BL/6 × 129 F1 hybrid mice (8–10 weeks; body weight 19–25 g) were used in this study. For implantation of electrodes in thalamocortical circuits, mice were anesthetized (ketamine/xylazine cocktail, 120, and 6 mg/kg, respectively, intraperitoneal) and then positioned in a stereotaxic apparatus. Two microscrew-type electrodes (chrome-plated stainless steel, 3 mm in length and 1 mm in diameter, Asia Bolt, Seoul, Korea) were fixed on the skull areas above motor-related frontal (M1; 0.74 mm anterior and 1.5 mm lateral to bregma) and somatosensory-related parietal (S1; 1.82 mm posterior and 3.0 mm lateral to the bregma) cortical regions, and two wire-type electrodes (Teflon-insulated tungsten wire, 76.2/114.3 μm in bare/coated diameter, A-M Systems, Sequim, WA, USA) were implanted in motor (VL; 1.06 mm posterior, 1.1 mm lateral and 3.5 mm ventral to bregma) and sensory (VPM; 1.82 mm posterior, 1.5 mm lateral and 3.7 mm ventral to bregma) nuclei of thalamus. For ground and reference, one screw electrode was fixed on the interparietal bone. Location of electrode placement is drawn in inset of Figure 1A.

To allow unobtrusive administration of anesthetic drugs, a polyethylene tube (10 cm in length, 15020/86 μm in outer/inner diameter; PE 100, Intramedic polyethylene tubing, Clay Adams, Sparks, MD, USA) was inserted into the abdominal cavity and securely positioned by suturing. The other side of tube was affixed to the EEG connector. The detailed procedure is described in Hwang et al. (2010). Animals were allowed to recover for at least one week, prior to experimental use. After experimentation, histology was performed to verify the correct location of LFP electrodes [Figure 1 in previous publication (Hwang et al., 2012)], and only the data from mice with confirmed tip positions (*N* = 10) were used for analysis.

### Forced walking test

The forced walking test (Figures 1A,B) was utilized to precisely identify the moments of loss and recovery of motion following administration of anesthetics. This procedure is described in detail in Hwang et al. (2010). Briefly, the animals were placed on a treadmill (LE8708, Panlab, Spain) and after a brief habituation period (~15 min), were forced to walk at a constant speed of 5 cm/s. A three-axis accelerometer (MMA7260Q, Freescale Semiconductor Inc., Austin, TX, USA) was incorporated into the EEG/LFP headmount to allow monitoring of both animal movement and posture. We installed 5 inch-height walls on the four sides of treadmill in order to keep the mouse on the treadmill. Depending on the mice, the habituation periods for rest and moving treadmill were different, but ~15 min and 5 min were required, respectively. After observation of stable and natural walking on the treadmill, 10 min of baseline was recorded in each animal, and then a ketamine/xylazine cocktail (120 and 6 mg/kg, respectively) was administered through the preinstalled tube into the abdominal cavity while the animals were in their constant walking state. After loss of motion, the animals stayed on the treadmill against the wall receiving a constant frictional tactile stimulation due to the moving lane. This tactile stimulation did not advance the waking time significantly compared to the righting reflex test [Figure 2 in Hwang et al. (2010)]. The time of administration (*t*_ADM_), moment of loss of motion (*t*_LOM_), and the moment of recovery of motion (*t*_ROM_) were determined from the magnitude and angle data of the accelerometer (Hwang et al., 2010).

### EEG and LFP acquisition

Both EEG and LFP activity were continuously recorded while the mouse was on the running treadmill. Recordings included a 10 min baseline (wake state) period on the treadmill just prior to ketamine/xylazine administration, and were continued throughout anesthesia (*t*_LOM_ → t_ROM_). Recordings also include a recovery period longer than 15 min starting after the animal showed movement again. The EEG and LFP were acquired with an analog amplifier (8–16C, Grass Technologies, West Warwick, RI, USA) and digitized with an analog-digital converter (Digidata 1440A, Molecular Devices, Sunnyvale, CA, USA) at a sampling frequency of 2000 Hz. The representative raw traces of the signals in the different epochs are shown in Figure 5 in Hwang et al. (2010). The signals were band-pass filtered with cut-off frequencies of 0.5 and 100 Hz (linear-phase Bessel filter). We normalized the signals with average power in the frequency range of 90–100 Hz to set the impedance levels of different types of electrodes to the similar levels. Gamma activity (30–50 Hz) was monitored throughout the measurement period and compared across all the stages of vigilance. The conventional frequency range of gamma oscillation is 30 to 70 Hz {Traub, 2010 #35}, but we focused the 40 Hz oscillations which have been proposed to be related to cognitive processing and to the temporal binding {Joliot, 1994 #36.}

### Estimation of drug concentration in the brain tissue

We estimated the anesthetic drug concentration in the brain using three-compartment pharmacokinetic model including abdominal, peripheral, and brain compartments. The pharmacokinetic equation for the ketamine concentration of the brain, *c*(*t*) has been derived in Supporting Information in Hwang et al. (2012), i.e.,

(1) $$\begin{array}{l} c(t)=\frac{k_{a}FD}{V_{1}}(\frac{\left(k_{21}-k_{a}\right)}{\left(\alpha -k_{a})(\beta -k_{a}\right)}e^{-k_{a}(t\;-\;t_{0})}\\ \;+\;\frac{\left(k_{21}-\alpha \right)}{\left(k_{a}-\alpha )(\beta -\alpha \right)}e^{-\alpha (t\;-\;t_{0})}+\frac{\left(k_{21}-\beta \right)}{\left(k_{a}-\beta )(\alpha -\beta \right)}e^{-\beta (t\;-\;t_{0})} \end{array}$$

where *t*_0_ is the drug administration time. *k*_*a*_ is the absorption rate in the abdominal compartment and *k*_21_ is the transfer rate constant from peripheral compartment to brain compartment. α and β are the rate constants for drug distribution into and elimination from the brain compartment as functions of rate constants between compartments. *D, F*, and *V*_1_ are the absolute amount, the bioavailability of ketamine, and the volume of brain compartment, respectively. These parameters were canceled out during normalization procedure to obtain *c*(*t*)/*c*(*t*_0_). *k*_*a*_ and α are inverse proportional to the drug absorption time and anesthesia time, which were estimated from the inverse of (*t*_LOM_−*t*_ADM_) and (*t*_ROM_−*t*_LOM_), respectively. The ranges of *k*_*a*_ and α are 0.71 ± 0.27 and 0.11 ± 0.03 min^−1^ (mean ± *SD*), respectively. The other decay rate, β and the rate constants, *k*_12_ and *k*_21_ are 0.00508, 0.0684, and 0.0262 min^−1^, respectively, which are determined from the time-concentration profile of ketamine (Leung and Baillie, 1989). The estimated ketamine concentration, *c* was normalized by its maximum value to yield a value between 0 and 1.

### Data analysis

#### Phase and amplitude of oscillations

To obtain the phase-amplitude relationship between SWA and gamma oscillations, the signals were filtered between 0.5–4 Hz for SWA and 30–50 Hz for gamma oscillations (400th order zero-phase FIR band-pass filters for both SWA and gamma). The instantaneous phase of SWA (ϕ_Slow_) was computed from the Hilbert transformation of the filtered signals and then divided into equal-sized 18 bins (20°/bin). Gamma amplitude in each bin was calculated as followings. First, we computed the absolute magnitude of gamma oscillation, and then obtained their envelope by interpolating the positive peaks. Secondly, we calculated the gamma amplitude in each bin by averaging the envelope within the corresponding bin.

#### Detection of on state during SWA

For the comparison of gamma power and synchrony across different behavioral states, data was sampled from three stable periods: movement artifact-free baseline period tasking the mice with forced walking (BASE), anesthesia state (ANES) where the induced slow oscillation pattern was steady, and recovered state (RECV) which was at least 10 min after *t*_ROM_; and two transitional periods: anesthetic induction period between *t*_ADM_ and *t*_LOM_ (LOM) and emergence period centered at *t*_ROM_ (ROM). The length of the sampled periods for each subject was fixed to the length of period between *t*_ADM_ and *t*_LOM_, which was 1.61 ± 0.61 min (mean ± *SD*) (Figure 1B).

In addition, gamma activity during anesthesia was sub-classified into ON and OFF state oscillations based on its relationship to the phase of SWA (Figure 1C). ON state and OFF state gamma oscillations were defined as gamma oscillation that occurred around the peak (90° ≤ ϕ_Slow_<270°) and the trough (−90° ≤ ϕ_Slow_<90°) of SWA.

#### Power and synchrony of gamma oscillations

In the analysis of temporal change of gamma power, the gamma power denotes the average power between 30–50 Hz where the power was calculated with fast Fourier Transform (FFT) in 10-s sliding windows with 9-s overlap. In comparison among the sample periods of different behavioral states, the gamma power within the periods of interests was calculated by the mean square of gamma amplitude for each period and normalized to the gamma power of BASE period. The gamma synchrony was evaluated by calculating the phase synchrony with phase-locking value (PLV), which is defined as

(2) $${\text{PLV}}_{xy}=\frac{1}{N}\left|\sum_{n\;=\;1}^{N}e^{i\Delta \phi_{xy}(t_{n})}\right|$$

where Δϕ_*xy*_≤ft(*t*_*n*_) is the phase difference between signals *x* and *y* at time *t*_*n*_and *N* is the number data points in the 10-s analysis window (sliding windows with 9-s overlap). The instantaneous phase of gamma oscillation was obtained by Hilbert transformation of the 30–50 Hz band-pass filtered signal (400th order zero-phase FIR band-pass filters). Friedman's ANOVA test and *post-hoc* Wilcoxon signed rank test with Bonferroni correction (5% significance level) was performed to check significance of difference among sampled states. All the analysis was performed by custom-scripted MATLAB (MathWorks, Natick, MA, USA) software.

## Results

### Changes in gamma power across ketamine/xylazine anesthesia

**Figure 2:**
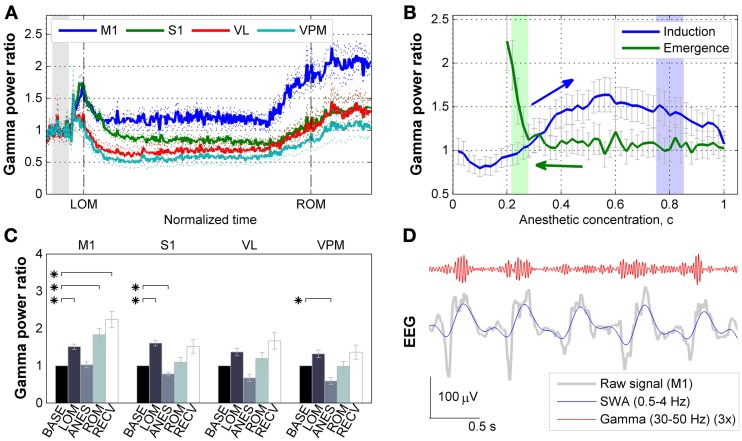
**Effects of ketamine/xylazine on gamma power. (A)** Subject-averaged temporal change of gamma (30–50 Hz) power with respect to normalized time window between *t*_LOM_ and *t*_ROM_ (dashed vertical lines, 33.23 ± 8.34 min from *t*_LOM_ to *t*_ROM_, mean ± *SD*). Gray shade indicates distribution of relative positions of *t*_ADM_ in reference to *t*_LOM_. Dotted lines for s.e.m. Each power was baseline corrected. **(B)** Subject-averaged change of gamma power in M1 with respect to anesthetic concentration, **(C)** estimated with three-compartment anesthetic concentration model. Gray whiskers for s.e.m. Zero anesthetic concentration corresponds to *t*_ADM_ and arrows indicate flow of time. Blue and green shaded patches indicate the distribution of *t*_LOM_ and *t*_ROM_ with corresponding concentrations of 0.80 ± 0.05 and 0.25 ± 0.03, respectively. **(C)** Change of gamma power at different brain regions for different behavioral states. Error bars indicate s.e.m. Asterisks indicate significant (*p* < 0.05, Friedman's ANOVA test and *post-hoc* Wilcoxon signed rank test with Bonferroni correction) difference from baseline. **(D)** Gamma oscillation superimposed on slow (0.5–4 Hz) oscillation during anesthesia. Gamma oscillation is exaggerated for the sake of clarity. BASE, baseline; LOM, loss of motion; ANES, anesthesia; ROM, recovery of motion; RECV, recovered state; M1, primary motor cortex; S1, primary somatosensory cortex; VL, ventral lateral thalamic nucleus; VPM, ventral posteromedial thalamic nucleus.

First we examined how gamma activity level changed across anesthesia and subsequent recovery. Following ketamine/xylazine administration, as the level of anesthesia deepened the oscillatory pattern observed in corticothalamic EEG/LFP recordings became more regular and showed an increase in slower frequency activity with a peak frequency at 1.5 Hz [Figure 4A in Hwang et al. (2010) and Figure 2C in Hwang et al. (2012)]. The ensemble averaged traces of gamma power of 10 mice are shown in Figure 2A with respect to a rescaled time which was divided by the time interval between *t*_ADM_ and *t*_ROM_. The average drug induction time (*t*_ADM_ to *t*_LOM_) was 1.61 ± 0.61 min and the average inactive time (*t*_LOM_ to *t*_ROM_) was 33.23 ± 8.34 min (*N* = 10, mean ± *SD*). These traces show a strong initial increase in gamma power just after *t*_ADM_ across all regions recorded which peaked just prior to *t*_LOM_. Gamma power was then observed to decrease during deep anesthesia, and then entered into the rising mode several minutes before *t*_ROM_. Gamma power for the primary motor cortex (M1) was maintained at a higher level during anesthesia compared to baseline, while in the primary somatosensory cortex (S1) and thalamic nuclei, gamma power levels fell below baseline during deep anesthesia.

The observed fluctuations in gamma power appear to be related to changes in the anesthetic concentration in the brain. In particular, the gamma power in M1 depicted the effect of anesthetic concentration showing an initial increase followed by decrease during induction period (Figure 2B). On the other hand, gamma power in M1 remained unvaried during emergence period, showing a sharp increase right before *t*_ROM_. Similar patterns of gamma power were observed in other brain regions recorded (data not shown).

Figure 2C provides the changes in gamma power in all corticothalamic brain regions recorded, across the different behavioral states associated with ketamine/xylazine anesthesia. Looking initially at the LOM period at the start of anesthesia, gamma power levels were increased compared to baseline across all brain regions. However, this increase was found to be statistically significant (*p* < 0.05) in the cortex, M1 and S1. During the period of steady anesthesia that followed (ANES), gamma was then observed to decrease to near or below baseline levels, with both the S1 and somatosensory thalamus (VPM) showing a significant (*p* < 0.05) decrease compared to baseline. For the ROM period, gamma power showed a rising trend for all regions, but only in the M1 did this rise reach statistical significance (*p* < 0.05). During the ensuing recovery period (RECV) gamma power level remained elevated. Again, this increase was statistically significant only in the M1 region (*p* < 0.05). Although the animals recovered head and limb movement after *t*_ROM_, our estimates of brain anesthetic concentration (see below) showed that the drug was not completely washed out by this time (Figure 2B). Thus, the elevated gamma power level during the recovery period (lasting for 31.52 ± 8.08 min after *t*_ROM_, mean ± *SD*) is considered to be a remnant effect of the anesthetic drugs.

### Gamma activity is modulated by the phase of SWA during ketamine/xylazine anesthesia

**Figure 3:**
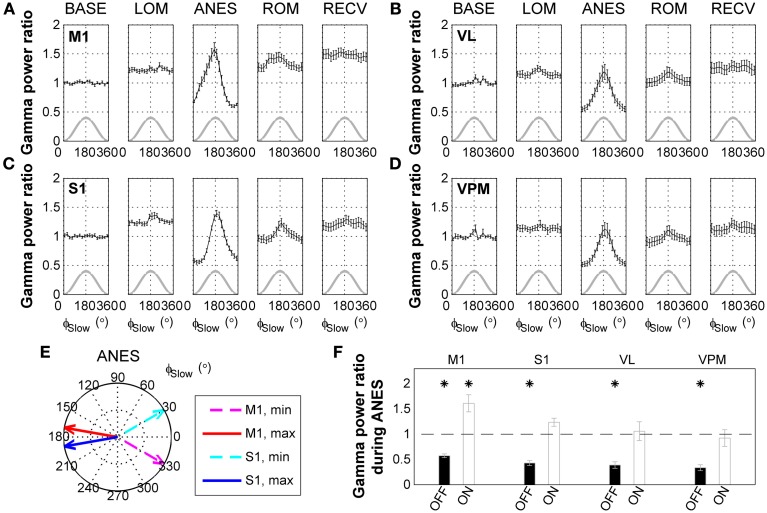
**State-dependent modulation of gamma activity during ketamine/xylazine anesthesia**. Modulation of gamma (30–50 Hz) activity by the phase of slow wave activity (0.5–4 Hz) at **(A)** M1, **(B)** VL, **(C)** S1, and **(D)** VPM across ketamine/xylazine anesthesia. Gamma power is normalized to baseline level and drawn with respect to the phase of slow oscillation (ϕ_Slow_). Gray lines for corresponding slow oscillation. Error bars, mean ± s.e.m. Gamma power is represented as a ratio to baseline gamma power level. **(E)** Different positions of maximal and minimal gamma activities between M1 and S1 cortical regions during ANES period. Arrows in polar coordinates indicate the phases of slow oscillation at which gamma power reaches its minimum (dashed) and maximum (solid), respectively. Angular coordinate is the phase of slow oscillation (zero for trough and 180 for peak). **(F)** Different behavior of gamma power during anesthesia state depending on the phase of slow oscillation. Gamma power during OFF state and ON state of the slow oscillation was significantly different from each other for all the regions (*p* < 0.05, Wilcoxon signed rank test). Horizontal dashed line indicates baseline level and asterisks indicate significant (*p* < 0.05, Friedman's ANOVA test and *post-hoc* Wilcoxon signed rank test with Bonferroni correction) difference from baseline. Error bars indicate s.e.m. BASE, baseline; LOM, loss of motion; ANES, anesthesia; ROM, recovery of motion; RECV, recovered state; M1, primary motor cortex; S1, primary somatosensory cortex; VL, ventral lateral thalamic nucleus; VPM, ventral posteromedial thalamic nucleus.

Interestingly, a comparison of raw surface EEG signals with SWA-band and gamma-band filtered signals recorded during anesthesia (Figure 2D) appeared to indicate that gamma activity is phase-locked with respect to the SWA. Thus, we next examined the phase-amplitude relationship between SWA and gamma oscillations across ketamine/xylazine anesthesia. As shown in Figures 3A–D, gamma amplitude varied depending on the phase of SWA during anesthesia with higher values occurring around ON state (around the peak of SWA) and lower values around OFF state (around the trough of SWA). This observation is in line with earlier findings by Chauvette et al. (2011) and Le Van Quyen et al. (2010). In addition, the phase of SWA at which gamma amplitude reached its extrema was observed to be different between different cortical regions during ANES period such that M1 changed in advance to S1 (Figure 3E). During stable anesthesia, the OFF state showed significantly decreased gamma power (*p* < 0.05) for all brain regions recorded compared to wake baseline, while during the ON state, baseline levels of gamma activity were observed in the S1, VL, and VPM regions, and actually showed a significant increase (*p* < 0.05) in M1 (Figure 3F).

### Cortico-cortical frontoparietal gamma synchrony is impaired during ketamine/xylazine anesthesia

**Figure 4:**
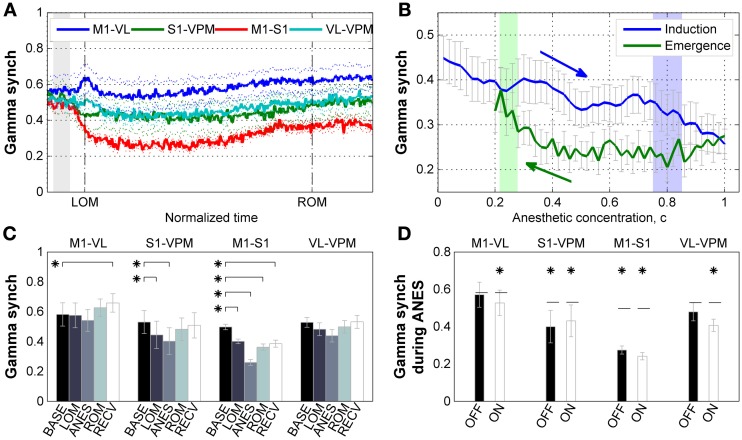
**Properties of gamma synchrony. (A)** Subject-averaged temporal change of gamma (30–50 Hz) synchrony with respect to normalized time window between *t*_LOM_ and *t*_ROM_ (dashed vertical lines, 33.23 ± 8.34 min from *t*_LOM_ to *t*_ROM_, mean ± *SD*). Gray shade indicates distribution of relative positions of *t*_ADM_ in reference to *t*_LOM_. Dotted lines for s.e.m. **(B)** Subject-averaged change of gamma synchrony between frontal-parietal cortical pair (M1-S1) with respect to anesthetic concentration, **(C)**. Gray whiskers for s.e.m. Zero anesthetic concentration corresponds to *t*_ADM_ and arrows indicate flow of time. Blue and green shaded patches indicate the distribution of *t*_LOM_ and *t*_ROM_ with corresponding concentrations of 0.80 ± 0.05 and 0.25 ± 0.03, respectively. **(C)** Change of gamma synchrony at different brain regions for different behavioral states. Error bars indicate s.e.m. **(D)** Different behavior of gamma synchrony during anesthesia state depending on the phase of slow oscillation. There was no significant difference between synchrony values during OFF state and ON state of the slow oscillation. Horizontal dashed lines indicate baseline level for each pair. Asterisks indicate significant (*p* < 0.05, Friedman's ANOVA test and *post-hoc* Wilcoxon signed rank test with Bonferroni correction) difference from baseline. Error bars indicate s.e.m. BASE, baseline; LOM, loss of motion; ANES, anesthesia; ROM, recovery of motion; RECV, recovered state. M1, primary motor cortex; S1, primary somatosensory cortex; VL, ventral lateral thalamic nucleus; VPM, ventral posteromedial thalamic nucleus.

We next examined how gamma phase synchrony was affected by ketamine/xylazine anesthesia between the corticothalamic regions examined. As shown in Figure 4A, a decline in synchrony between M1 and S1 was observed following administration of anesthesia, while a change in synchrony between the other recorded corticothalamic regions was not prominent. Examination of the relationship between gamma synchrony in cortical pair and estimated brain anesthetic concentration (Figure 4B) showed a monotonic decrease in synchrony during induction of anesthesia. The sharp increase of gamma power during induction period was absent in cortical gamma synchrony. During emergence, the corticocortical gamma synchrony initially remained unvaried, and then started to restore just prior to *t*_ROM_.

Figure 4C shows the changes of gamma synchrony as the ANES changes. During the initial stage of anesthesia (LOM) a significant reduction in gamma synchrony (*p* < 0.05) can be observed between M1 and S1 pair. This reduction increased during stable anesthesia (ANES), and was progressively restoring but not fully restored to the baseline level as the mice emerged (ROM) and recovered from anesthesia (RECV). A similar state-dependent change in gamma synchrony was also found in the somatosensory electrode pair (S1 and VPM), showing a significant reduction in synchrony during both initial and stable anesthesia (*p* < 0.05), and restoration of synchrony to baseline levels during emergence and recovery from anesthesia. Synchrony bewteen motor-related electrode pair (M1 and VL) and thalamic electrode pair (VPM and VL) showed no significant change in gamma synchrony across anesthisa. However, a significant increase was observed during the RECV period for the motor-related pair (*p* < 0.05). Unlike our analysis of gamma power, gamma synchrony showed no significant slow wave phase-locked modulation during anesthesia and had reduced or similar value compared to baseline (Figure 4D). Thus, there exists a dissociation between gamma power and synchrony such that increased gamma power is not always accompanied by increased gamma synchrony.

## Discussion

In this study we examined the behavior of gamma oscillatory activity throughout anesthesia-induced unconsciousness and consecutive recovery. Under ketamine/xylazine anesthesia, gamma power in corticothalamic regions was found to be initially increased following drug administration, then fall to near or below baseline (wake) levels corresponding to LOM (Hwang et al., 2010). Gamma power remained at this level throughout stable anesthesia, and was again elevated just prior to ROM. Despite the persistence of gamma throughout anesthesia, we observed that the gamma activity observed during ketamine/xylazine induced anesthesia was nested in the ON state of SWA. Further, the phase synchrony of gamma activity during anesthesia was decreased compared to that observed during wake significantly between cortical brain regions related to somatosensory and motor function.

Previous studies have suggested that the ON state of SWA perhaps represent fragmented periods of wake state (Destexhe et al., 2007; Constantinople and Bruno, 2011). However, the recurrent hyperpolarization of SWA may discourage consciousness by interfering with active neural processing by impairing the reliable transfer of sensory information in the cerebral cortex. Our results supports the hypothesis that reduced long-range gamma synchrony between brain areas during ON state of oscillation also can be one of mechanisms which make difference in conscious experience during period of reduced consciousness, such as anesthesia and sleep, even though ON state and wake state have similar electrophysiological features of increased gamma power.

The ability of multiple cortical regions to interact in a rapid and effective manner has been suggested to represent a key requirement for consciousness (Tononi, 2005). Thus, the precise synchrony of activity across distributed cortical networks is most responsible for conscious processing (Schroeder and Lakatos, 2009). Our results are consistent with this idea, and suggest that measures of gamma synchrony are more informative about the level of consciousness than the change of gamma power at local brain regions. This implication is underpinned by the observation of significant decrease in gamma synchrony between VPM and S1 contrary to the insignificant change in gamma power of VPM during deep anesthesia possibly due to the tactile stimulation given to the animals lying on the constantly moving treadmill. In addition, our observation is consistent with previous reports that a breakdown of feedback information flow between anterior-posterior regions of the brain can be observed during unconscious states induced by various anesthetics (Hudetz, 2006). Such dissociation between gamma power and synchrony tells that increase of gamma power does not necessarily enhance synchrony between LFP signals in macroscopic brain regions, which was proposed as a mechanism for information integration (Gray and Singer, 1989; Engel and Singer, 2001) between brain regions.

In contrast to unconsciousness induced by most hypnotic anesthetics, the unconsciousness induced by ketamine is associated with more active EEG patterns (Brown et al., 2011). The effect of ketamine/xylazine on gamma power observed here is likely resultant from the previously described NMDA receptor antagonist mediated disinhibition of cortical circuit activity (Homayoun and Moghaddam, 2007; Pinault, 2008; Lazarewicz et al., 2010; Carlen et al., 2012). Specifically, we assume the increased gamma activity observed just after *t*_ADM_ and prior to *t*_ROM_ occurs as the anesthetic (ketamine) is present in the brain at relatively low sub-anesthetic levels. Increased gamma activity with acute administration of ketamine has been shown to result in disinhibition of pyramidal neurons, likely due to preferential antagonism for NMDA receptors in parvalbumin-containing interneurons (Grunze et al., 1996; Homayoun and Moghaddam, 2007; Carlen et al., 2012). Such disinhibition would promote spontaneous firing of pyramidal neurons in a less organized fashion, which is reflected in the increased power but reduced synchrony of gamma band after ketamine/xylazine administration. As the ketamine concentration in the brain increases to anesthetic levels, it also begins to act on NMDA receptors in pyramidal neurons, relieving this disinhibition, resulting in the observed drop in gamma following LOM, which persists through ANES.

Previous studies regarding gamma oscillatory activity during states of impaired consciousness (sleep and anesthesia) have produced contradictory results. EEG studies in humans and animals have shown that gamma power and coherence (30–58 Hz) were lowest during natural slow-wave sleep compared to wake state (Maloney et al., 1997; Cantero et al., 2004). More recently, Le Van Quyen et al. (2010) found that gamma power (40–120 Hz) was highest during slow wave sleep in humans and Chauvette et al. (2010) reported that gamma band power (30–100 Hz) was even higher during ketamine/xylazine anesthesia compared to slow wave sleep in cats. These conflicting observations might arise from difference of natural sleep and anesthesia (Chauvette et al., 2010), but it should be also considered that naïve comparison of average gamma power by averaging ON and OFF state oscillation altogether may underestimate the results due to modulation of gamma activity by the phase of SWA. As suggested by Steriade (2006), neuronal activity during both conscious and unconscious states are defined by complex and distinct patterns of oscillatory activity. While gamma frequency activity is generally regarded as a defining characteristic of activity observed during conscious active brain states, such activity can also be found during periods of reduced consciousness. As suggested by Alkire and Miller (2005) and supported by our findings, increased brain activity alone does not provide a reliable means to measure consciousness. State-dependent changes of gamma power and synchrony at somatosensory and motor-related cortical and thalamic regions revealed that the change of long-range synchrony between the cortical regions well reflected changing depth of anesthesia. While power of gamma oscillation superimposed on ON state of slow oscillation was found not different from baseline level, synchrony of gamma oscillation was significantly decreased at this state for all pairs. Our finding implies that lacking synchrony between brain regions might be one of mechanisms impeding conscious experience during depolarized ON state which has been reported to have electrophysiological features similar to wake state.

### Conflict of interest statement

The authors declare that the research was conducted in the absence of any commercial or financial relationships that could be construed as a potential conflict of interest.
